# Towards AI-driven longevity research: An overview

**DOI:** 10.3389/fragi.2023.1057204

**Published:** 2023-03-01

**Authors:** Nicola Marino, Guido Putignano, Simone Cappilli, Emmanuele Chersoni, Antonella Santuccione, Giuliana Calabrese, Evelyne Bischof, Quentin Vanhaelen, Alex Zhavoronkov, Bryan Scarano, Alessandro D. Mazzotta, Enrico Santus

**Affiliations:** ^1^ Women’s Brain Project (WBP), Gunterhausen, Switzerland; ^2^ Dermatology, Catholic University of the Sacred Heart, Rome, Italy; ^3^ UOC of Dermatology, Department of Abdominal and Endocrine Metabolic Medical and Surgical Sciences, A. Gemelli University Hospital Foundation-IRCCS, Rome, Italy; ^4^ Department of Chinese and Bilingual Studies, The Hong Kong Polytechnic University, Hong Kong, China; ^5^ Department of Translational Medicine and Surgery, CatholicUniversity of the Sacred Heart, Rome, Italy; ^6^ Insilico Medicine Hong Kong Ltd., New Territories, Hong Kong SAR, China; ^7^ Department of Digestive, Oncological and Metabolic Surgery, Institute Mutualiste Montsouris, Paris, France; ^8^ Biorobotics Institute, Scuola Superiore Sant’anna, Pisa, Italy; ^9^ Bayer USA, New Jersey, United States

**Keywords:** artificial intelligence, machine learning, biomarkers, feature selection, deep aging clock, longevity medicine

## Abstract

While in the past technology has mostly been utilized to store information about the structural configuration of proteins and molecules for research and medical purposes, Artificial Intelligence is nowadays able to learn from the existing data how to predict and model properties and interactions, revealing important knowledge about complex biological processes, such as aging. Modern technologies, moreover, can rely on a broader set of information, including those derived from the next-generation sequencing (e.g., proteomics, lipidomics, and other omics), to understand the interactions between human body and the external environment. This is especially relevant as external factors have been shown to have a key role in aging. As the field of computational systems biology keeps improving and new biomarkers of aging are being developed, artificial intelligence promises to become a major ally of aging research.

## 1 Introduction

The process of aging is known to be dependent upon the interaction of different factors, such as the genome content of an individual, lifestyle factors, environmental interaction, and health facilities available to the individual (Newman and Murabito, 2013; Partridge et al., 2018; Singh et al., 2019). Increased lifespan and age represent the exceptional survival, maintenance of good health as compared to peers, delayed onsets of age-dependent diseases, and extreme phenotype of individuals (Kaeberlein, 2018; Pignolo, 2019).

Previous works have emphasized how modern Artificial Intelligence (AI) is already playing an important role in speeding up decision-making in medical sciences by means of advanced machine learning (ML) algorithms. For example, it is revolutionizing the drug discovery process, saving money and time (Kelemen et al., 2008), as it is already being used to create the structure of new drugs depending on the specific structure of the target disease-causing compound [see (Santus et al., 2021) for an overview]. In life sciences, the generation of high-throughput data such as proteomics, genomics, chemoproteomics and phenomics combined with the recent development of AI technologies (Alberghina and Westerhoff, 2007; Santus et al., 2021) and the availability of increasingly powerful computational resources allow the deployment of complex ML methods to preprocess massive amounts of data, integrate different input modalities and identify insightful correlations (Kulaga et al., 2021).

**FIGURE 1:**
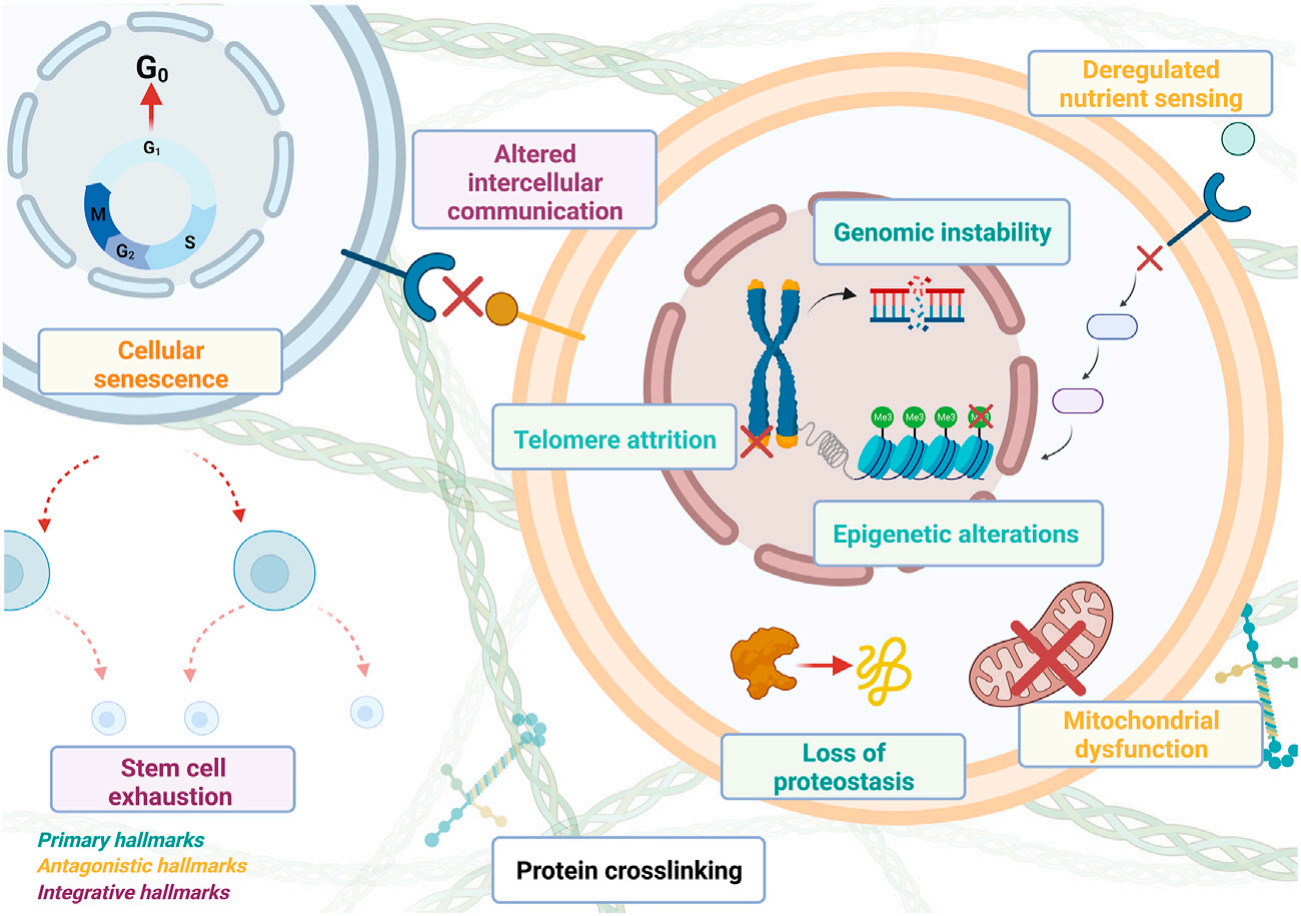
Summary of the hallmarks of aging. Primary hallmarks of aging, the primary causes of cellular damage, include genomic stability, epigenetic alterations, loss of proteostasis, and telomere attrition. Antagonistic hallmarks (this refers to factors that originated from body responses to the damage itself but end up exacerbating it) include mitochondrial dysfunction, deregulated sensing, and cellular senescence. Integrative hallmarks of aging (that result from the cumulative action of the previous two groups and are the main determiners of the functional decline) include stem cell exhaustion and altered intercellular communication. Each of these hallmarks has been the focus of intensive research to understand their involvement in the decline of biological functions. ML/AI technologies are used to deepen our understanding of the many components that are involved. This knowledge can help to improve not only our understanding of these mechanisms taken separately but also how the interplay between them unfolds.

The increasing availability of biological data of all types has contributed to greatly improve our understanding of the human body and the systemic nature of biological systems in general. This was accompanied by a conceptual change within biology with the transition from a qualitative, reductionist, structural and most of the time static description to a more systemic description in terms of functional and dynamical properties (Barabási and Oltvai, 2004; Bruggeman and Westerhoff, 2007; Liang et al., 2011). Now, biological entities are more and more often described as dynamical systems made of a multilayered hierarchy of sub-systems containing large numbers of highly connected components. ML techniques are well adapted to discover not only correlations but also causal relations between data to identify key interactions and key regulators that must be integrated into a model to uncover mechanisms that can explain the emergence of specific biological functions. In the context of aging research, there are several mechanisms; called hallmarks of aging (*cf.* Figure 1), that have been identified as playing a central role in the onset and propagation of aging. Understanding the causal relationships taking place within biological systems is a prerequisite to build dynamical, i.e., kinetic, models that can be used to simulate the integral response of a biological system during its development, the progression of a disease, or during pharmaceutical interventions. Dynamic models have been designed, for example, to describe the effects of inflammation, senescence, apoptosis, oxidative stress, accumulation of mutations and DNA damages, cell cycle deregulation, mitochondrial dysfunction, and telomere shortening (Auley et al., 2017). Interestingly, many of these mechanisms have been associated over time with specific pathologies, called aging-related-diseases (ARDs), which commonly appear when individuals get older. For example, various types of cancers are identified as ARDs and their origin is assumed to be connected to genomic instability and decreased capacity for DNA repair, two characteristics of both cancer and aging (Maslov and Vijg, 2009). Many of the mechanisms triggering ARDs have been used to elaborate specific theories of aging, which propose to explain the onset and propagation of aging from a set of molecular mechanisms and leading functions associated with ARDs.

The surge in aging research and associated R&D investments witnessed during the recent years should be put in perspective with the continuous increase in human life expectancy observed over the last decades which has significant long term social impacts and economic consequences. One estimated that over 700 million people were over 65 years old in 2019, a number that might double by 2050 (Prince et al., 2016). Hopefully, thanks to intense scientific research, we are continuously improving our understanding of the intertwined biological processes behind aging. This valuable knowledge combined with the possibilities created by technological developments can be used to develop novel treatments which are much needed in societies where the rise in human longevity is often accompanied with an increased burden of chronic diseases and ARDs including cardiovascular diseases, cancer, and neurodegenerative diseases such as Parkinson disease (PD) and Alzheimer’s disease (AD) (Partridge et al., 2018). Beside the fact that these ARDs result in reduced quality of life of the elderly population, they also present a healthcare and socioeconomic challenge. Many countries facing a continuously aging society have already embraced this challenge by initiating ambitious healthcare development programs and adaptation plans to be able to cope with these unavoidable societal trends. In this context, ML and AI combined with big data and other novel technologies can be deployed to monitor disease patterns within a population, develop adapted geriatric care systems, prioritize, and optimize drug development and design appropriate public health policies to foster healthy aging habits and improved lifestyle among all segments of the populations (Fang et al., 2020).

Aging research and its offspring, longevity research are two very active and rapidly evolving fields. In the present contribution, we propose to discuss a subset of studies which, in our opinion, should provide interested readers and researchers with a broad overview of how aging, when considered from a mechanistic perspective, can be investigated from different viewpoints using classical computational methods combined with ML/AI approaches, leveraging the opportunities offered by the continuously growing sets of health and biological data. There is a tremendous variety of questions and topics of interest to be covered and a large diversity of research methods deployed to investigate them. Scientific studies can investigate biological mechanisms at a fundamental level to clarify their links with the onset of ARDs. In several cases, the authors use the newly acquired knowledge to develop aging clocks. Other research works utilize the already established knowledge of aging mechanisms and associated signaling pathways to identify through computational means potential novel therapeutic targets. In some cases, such analysis proposes a complete workflow with either *in vitro* and/or *in vivo* validation to support the computational findings. These studies are also sometimes complemented with virtual screening experiments to identify compounds with suitable drug-like properties which could constitute the basis of novel therapies. Thus, to structure the discussion, we decided to follow the taxonomy introduced in the classical work by (López-Otín et al., 2013) and organized this article according to the three main areas of intervention of AI technologies. An outline of the article is as follows. Section 2 covers the primary hallmarks of aging, that is, the primary causes of cellular damage, Section 3 focuses on antagonistic hallmarks, namely, those factors that originated from body responses to the damage itself but end up exacerbating it. Section 4 discusses studies interested in the integrative hallmarks which result from the cumulative action of the previous two groups and are the main determiners of the functional decline. We discuss several possible clinical applications in Section 5 and end up with a conclusion in Section 6.

## 2 Primary hallmarks

### 2.1 Genomic instability

Genomic instability is the growing tendency of cells to accumulate mutations both in nuclear and mitochondrial DNA, and it is considered as one of the primary hallmarks of the aging process (López-Otín et al., 2013; Laffon et al., 2021). The micronucleus (MN) test and its evolution, the cytokinesis-block micronucleus (CBMN) test, are the most used methodologies to evaluate genomic instability and quantify DNA damage in different tissues. Therefore, their results can be used as biomarkers of genomic instability in aging (Laffon et al., 2021).

Such tests allow measuring the number of micronuclei in several types of cells (Laffon et al., 2021; Wills et al., 2021). Micronuclei are chromosome-derived structures, surrounded by a membrane, that arise from fragments of acentric chromosomes or from entire chromosomes that fail to bind to the mitotic spindle and to segregate properly in the daughter cells (Fenech et al., 2016). The frequency of micronuclei in peripheral lymphocytes has been shown to increase by age groups, with a frequency ratio of 1 in children (<10 years old) and a frequency over 2 in elder people (>70 years old) (Bonassi et al., 2001).

**FIGURE 2:**
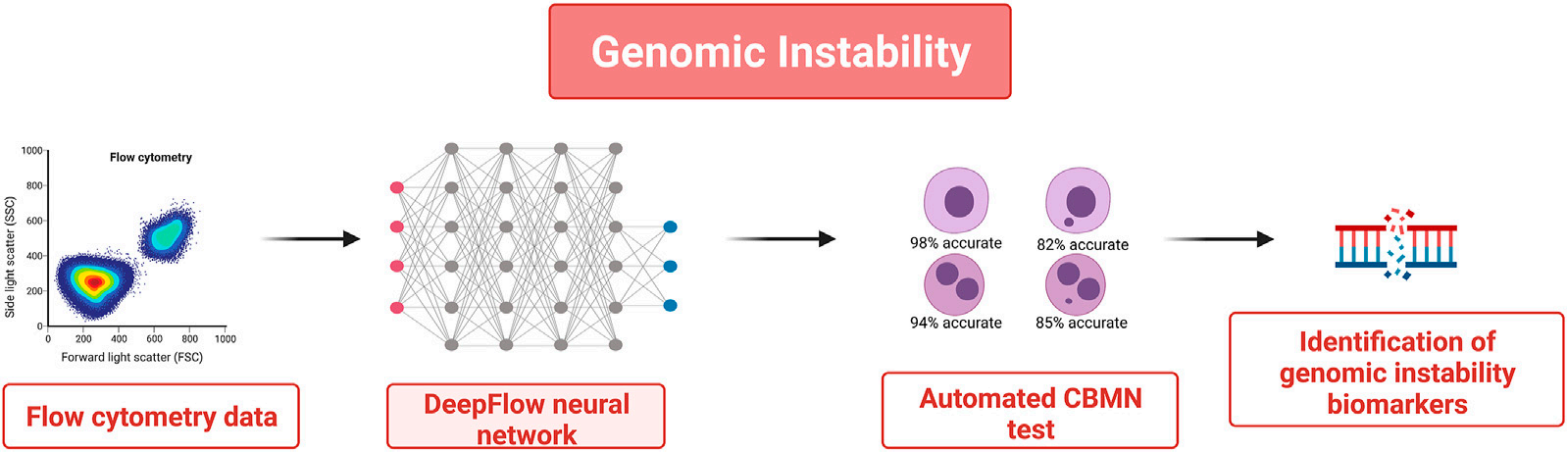
AI-based automated CBMN test to quantify genomic damages in tissues. Thousands of images from flow cytometry data were used to train a DL-based image classifier. This allows an automated scoring of the evaluation of the number of micronuclei while reducing time overload and false positives.

However, a limitation of the CBMN test is that it requires users’ manual scoring which makes it time-consuming and subjective, with very low specificity and a high number of false positives (Wills et al., 2021). For these reasons in the last few years the CBMN test was associated with imaging flow cytometry and deep learning (DL) algorithms to automatize and accelerate the procedure (see Figure 2). In (Wills et al., 2021), the authors developed an automated image classification for the CBMN assay by training and cross-validating the “DeepFlow” neural network with data obtained in several laboratories that used different techniques for sample preparation, cytometer calibration, and image acquisition. They trained the system with tens of thousands of images from two laboratories and then tested it with the images of a third one. The “DeepFlow” neural network took less than 2 min to score thousands of images with an accuracy of 98% in mononucleate cells, 82% in mononucleate cells plus MN, 94% in binucleate cells, and 85% in binucleate cells plus MN. Interestingly when evaluating the cases in which the result of the software was different from the human-scored one it was found out that the software was sometimes more valid because it can classify the MN according to its area and to the size of the nucleus of the relative cell. Thus, in this case, integrating AI technology contributed to improve not only the average speed but also the accuracy of the procedure.

A similar automated method was developed by combining imaging flow cytometry with custom-designed software and AI to score the micronuclei in a 3D skin-based model (Allemang et al., 2021). The evaluation of reconstructed skin allows using cells that are naturally more exposed to genotoxic substances, and AI makes it a completely automatic method devoid of subjective scoring. By further training these systems or similar ones, even without user configuration, it will be possible to eliminate the variability of the CBMN test and significantly reduce the associated costs. In this way, the results of the CBMN test will be more accurate biomarkers of genomic instability. Although, until now, this type of test has been primarily used to evaluate genotoxicity, it would be possible to assess aging in the same way and to estimate the biological age of a patient.

### 2.2 Epigenetic alterations

The term epigenomics refers to mechanisms regulating genome activity independently of changes to DNA sequence (Felsenfeld, 2014). These mechanisms that induce reversible changes can be classified into four categories: remodeling of chromatin conformation, DNA modifications such as DNA methylation, histone post-translational modifications, and RNA-centered mechanisms (including non-coding RNAs and microRNAs) (Pagiatakis et al., 2021). Epigenetics is the main mechanism by which environmental factors such as stress, physical activity, diet but also alcohol consumption influence gene expression. Epigenetic mechanisms can also be modulated by physiological and pathological stimuli (Pagiatakis et al., 2021). Although epigenetic modifications attracted interest for their involvement in aging and in the onset of ARDs, they are also essential for development processes such as tissue and organ formation.

The investigation of epigenetic mechanisms, epigenetic changes and their relationships with aging must begin with the prediction of epigenetic-relevant features such as epigenetic sites and genetic alterations. Despite some successful applications of ML/AI for epigenome mapping (Angermueller et al., 2017) and for the identification of susceptible epimutation sites in the genome (Haque et al., 2015), it should be emphasized that these essential steps already present specific challenges. Indeed, experimental protocols to study epigenetic mechanisms are typically expensive to implement and to some extent ML/AI methods such as active learning must be deployed to reduce the expense of generating epigenetic data. Furthermore, analyzing epigenetic data can be cumbersome because like other biological datasets, raw epigenetic data are typically high dimensional, but the occurrences of interest, i.e., epigenetic marks and epimutation sites, are difficult to find. For instance, DNA methylation data usually contain a few differentially methylated DNA regions (DMR) and many non-DMR sites, although both are described with many DNA sequence and genomic features (Holder et al., 2017). In fact, the number of genomic features within epigenetic data is huge, and the selection of the most relevant ones that could be used to locate epigenetic sites requires specific approaches (see below for an example). In this context, ML/AI techniques are necessary not only to identify the regions of the genome where the epigenetic changes of interest could occur and that are susceptible to epimutations, but also to preprocess and carefully annotate epigenetic data prior to any analysis. Workflows used to that end combine techniques for feature generation and selection, and techniques to deal with the specific characteristics of epigenetic data. Concretely, ML/AI is commonly used to help define the most relevant genomic features. Moreover, imbalanced class learning has proven to be useful to compensate for the relatively low occurrence of relevant epigenetic events.

**FIGURE 3:**
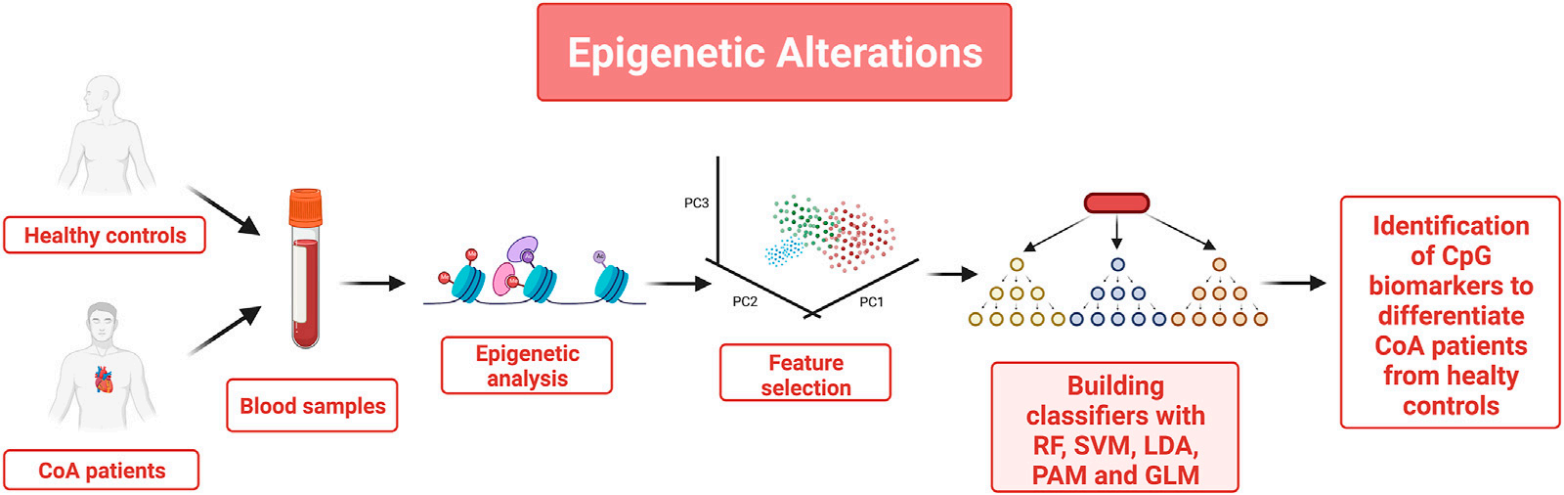
Blood samples were utilized to identify epigenetic changes that allow distinguishing healthy controls from patients with coarctation of the aorta (CoA). Cytosine nucleotide (CpG) methylation changes obtained from Genome-wide DNA methylation analysis were selected as mean features. Only the probes with statistically significant methylation differences combined with a sufficiently high methylation fold change were kept as features and Multivariate approaches such as principal component analysis (PCA) and partial least squares-discriminant analysis (PLS-DA) were used to confirm that these features could accurately distinguish the CoA samples from the controls. 5 classifiers were built and trained using these features. Features with the highest predictive capabilities are potential novel biomarkers candidates for CoA.

An example of how epigenetic marks can be selected and used to characterize a disease state is shown in (Bahado-Singh et al., 2022) where the authors determined whether epigenetic changes occur in patients with Coarctation of the aorta (CoA) (see Figure 3). CoA is a congenital heart defect that might have epigenetic origins because prior studies showed that significant methylation changes were found in the DNA of newborns with CoA. Thus, the authors decided to use Cytosine nucleotide (CpG) methylation changes in samples from patients with CoA and healthy patients (obtained from Genome-wide DNA methylation analysis of 24 newborn blood DNA with CoA cases and 16 unaffected controls) to build several classifiers to distinguish CoA samples from controls and identify epigenetic patterns specific to CoA. Feature selection was carried out using the probes with statistically significant methylation differences combined with a sufficiently high methylation fold change in CoA compared to controls. Multivariate approaches such as principal component analysis (PCA) and partial least squares-discriminant analysis (PLS-DA) confirmed the possibility to accurately segregate or differentiate the CoA group from controls based on CpG methylation levels. Different methods were used to build the classifiers: random forest (RF), support vector machine (SVM), linear discriminant analysis (LDA), prediction analysis for microarrays (PAM), and generalized linear model (GLM). Features with the highest predictive powers for the various classifiers were characterized by highly statistically significant methylation changes in several CpG loci in CoA relative to controls. To interpret these results from a biological viewpoint, further analysis was performed using Ingenuity pathway analysis to link key features to disease pathways associated with CoA. Furthermore, PCA and PLS-DA also showed that using a limited number of “principal components” (CpG markers) was enough to differentiate the CoA from the unaffected control groups. Those findings pave the way to the identification of novel epigenetic biomarkers for CoA.

The integration of systems biology, big data science and AI/ML can be a successful strategy to elucidate still largely unknown epigenetic mechanisms involved in aging and in ARDs. Recently, ML/AI has been successfully applied to analyze omics and clinical data gathered in epigenetic studies (Oh et al., 2015; Ladd-Acosta et al., 2016; Holder et al., 2017). These applications also included the detection of DNA methylation characterizing specific diseases and aging related pathologies (Crowgey et al., 2018). Another study focused on the identification of correlations among methylation marks and found that different methylation profiles exist for different diseases as well as for tissues of different types (Luedi et al., 2007). The findings supporting that aging and the onset of ARDs are often associated with specific and reproducible changes across DNA methylation sites were exploited to develop a class of biomarkers of aging usually known as epigenetic clocks. These clocks function by integrating information about DNA methylation sites known to be correlated with chronological age across multiple tissue types and different populations. These epigenetic clocks have been shown to be able to predict the possible onset of ARDs such cardiovascular diseases and different types of cancers (Bocklandt et al., 2011; Hannum et al., 2013; Horvath, 2013; Weidner et al., 2014). Other recent examples of epigenetic studies using ML/AI include the classification of prostate cancer (Aref-Eshghi et al., 2018) and heart disease (Dogan et al., 2018).

A point worth mentioning is that genetic and epigenetic studies are still generally accomplished independently and as a result, physiological relationships between genetics and epigenetics in diseases remain poorly understood (Hamamoto et al., 2019). Studies revealed that there is a degradation of functional transcriptional networks correlated with an increase in heterogeneity between the epigenome and the transcriptome during aging (Hernando-Herraez et al., 2019). Epigenetic alterations have also been shown to impact genome stability and promote genetic sequence mutations (Skinner et al., 2015; McCarrey et al., 2016). Thus, to improve our understanding of genetic variations and epigenetic deregulations, multimodal analyses of big omics data should be deployed using AI platforms that allow integration between genetics and epigenetics.

### 2.3 Telomere attrition

Telomeres are sequences of tens of kilobases formed by the repetition of six nucleotides associated with protective proteins located in the terminal regions of chromosomes. Telomeres have two main functions: to prevent DNA repair and recombination complexes from recognizing the linear ends of chromosomes as broken ends, and to protect the gene content of chromosomes from degradation due to the progressive shortening of DNA following replication (Blackburn et al., 2015). In fact, the DNA replication machinery fails to copy the final nucleotides of the chromosome causing a shortening of the telomeres. In specific types of cells, the telomerase, a reverse transcriptase, is active to prevent telomere shortening and maintain their length (Hou et al., 2017). When the telomere length reaches a certain threshold, known as Hayflick limit, the cell cycle stops, and the cell becomes senescent (Liu et al., 2019). Therefore, telomere shortening is associated with senescence and aging (López-Otín et al., 2013). Numerous meta-analyses have been conducted in recent years, especially to decipher the relationship between telomeres and environmental factors: those are very useful tools that allow increasing the samples used in statistical surveys but suffer from deep methodological differences (Wang et al., 2018).

The results of the meta-analyses suggest that telomere shortening is associated with increased mortality risk in the general population. They also provide insights on the impact of the environment on telomere shortening. For instance, several studies show that the Mediterranean diet and the absence of cigarette smoking are correlated with longer telomere length, while the relationship with physical activity is not clearly established (Arsenis et al., 2017; Astuti et al., 2017; Wang et al., 2018; Canudas et al., 2020). In all the above-mentioned studies, the authors suggested that larger-scale analysis and clinical trials would be necessary to confirm these conclusions with greater certainty.

Currently, the leukocyte telomere length is used as a biomarker for healthy aging, despite the fact that there are many inconsistent and contradictory data relating to its effectiveness (Hartmann et al., 2021; Vaiserman and Krasnienkov, 2021). In fact, as previously mentioned, there are environmental factors that contribute to the telomere shortening and it has been shown that the length of the telomeres fluctuates by 2%–4% monthly (Galkin et al., 2020). Therefore, without adequately accounting for most of the variables involved in the shortening of telomeres and in the oscillation of their length, it remains difficult to use this measure as a reliable biomarker.

Bioinformatics and deep mining approaches can be useful for better understanding the biological interactions of telomeres and telomerase. For example, (Hou et al., 2017), analyzed numerous databases with multiple bioinformatics approaches to map the interactions of telomerase reverse transcriptase (TERT, one of the two main components of telomerase) with other proteins and its function in different biological pathways. With similar bioinformatics approaches it will be possible, thanks to data already available, to obtain new information on the enzymes involved in the shortening of telomeres. A better understanding of the phenomenon of telomere attrition will make possible using this parameter as a reliable aging biomarker although recent articles and reviews have suggested that telomere attrition could just be used inside a panel together with other aging factors to improve the efficacy in the assessment of biological age (Galkin et al., 2020; Vaiserman and Krasnienkov, 2021).

### 2.4 Loss of proteostasis

Protein homeostasis, or proteostasis, refers to the balance that must be maintained between the newly folded proteins and the degradation of the superfluous, older proteins with the aim to prevent protein misfolding and accumulation of protein aggregates. Proteostasis is regulated through a complex network comprising molecular chaperones, proteolytic machinery, and their regulators (Hipp et al., 2019). The EU-funded project PROTEOSTASIS (Cell-type-specific modulation of protein homeostasis in health and disease) has investigated the proteostasis mechanisms and found that the identity and concentration of chaperones and proteostatic machinery are highly cell-type specific. Further investigation of the capacity of proteostasis in different tissues under various physiological and stress conditions shows that there is a strong decline in folding capacity in the transition to adulthood, followed by the loss of protein solubility and the accumulation of aggregates (when they exceed the physiological concentration). These results led to the conclusion that proteostasis dysregulation is the main cause of age-related protein misfolding diseases. From a broader perspective, it is important to develop methodologies to consider all the proteins, their different interactions, and the ways they change their expression through aging.

**FIGURE 4:**
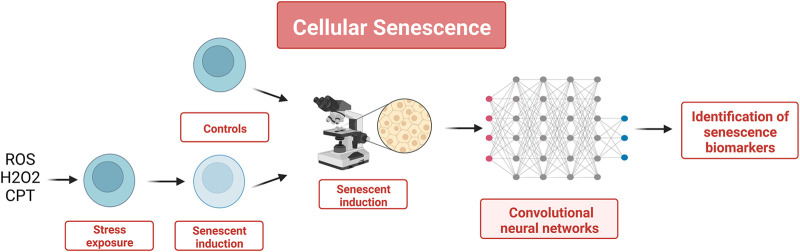
To identify novel aging-related proteins, the full set of human proteins was extracted from the Swiss-Prot database. Already known aging related proteins listed on the GenAge database were used as instances of the aging-related class. UniProt, Gene Ontology and GeneFriends databases were used to extract 21,000 protein characteristics that were subsequently used as features to train 3 classifiers to identify proteins likely to be associated with aging.

To obtain a better picture of which proteins intervene in the repair and maintenance mechanisms such as autophagy for instance which is well-known for being affected as individuals age, the authors of a recent study (Kerepesi et al., 2018) used ML methods to build classification models using multiple protein features to identify new aging-related proteins that have a particularly prominent role in repair and maintenance mechanisms (see Figure 4). Data used for this study included the set of aging-related genes available in GenAge, a manually curated database made of 305 aging-related genes. The corresponding proteins were used as instances of the aging-related class. All other human proteins in Swiss-Prot, were used as the instances of the non-aging-related class. Multiple features were extracted from the data. In total 21,000 protein features were extracted from different databases. This included co-expression and protein-protein interaction features such as the number of aging-related neighbors and several other topological properties of the protein-protein interaction networks associated with the selected proteins. Three ML methods (XGBoost, logistic regression (LR), and SVM) were deployed to build classifiers that could distinguish aging-related proteins from the non-aging-related ones. In the second part of the study, the trained models were utilized to predict new aging-related proteins. The score associated with the proteins by the classifiers gives an insight on the role of a protein in the aging process. To identify the most important aging-related features of human aging-related proteins, XGBoost feature selection capabilities were used to identify the top 36 protein features. A reduced and more easily interpretable model based on these 36 features was designed and demonstrated high prediction performance. It was used to select a list of the twenty new most relevant aging-related proteins. This study showed how the most relevant protein features can provide an insight into the regulation of the aging process.

Proteins found within human plasma are another unvaluable source of information to be used to characterize and quantify the onset and propagation of aging. Intensive research has been undertaken to systematically identify proteins whose expression shows strong patterns of changes with age and that could constitute a reliable proteomic signature of the phenomenon. Proteins found in plasma are attractive for this endeavor because they affect phenotypes and are directly involved in the dynamics of signaling pathways regulating many of the physiological manifestations of aging. From this viewpoint, proteomic signatures are even more advantageous than epigenetic signatures for instance because the effects of molecular changes such a DNA methylations and other epigenetic changes are not always very well understood. In Tanaka et al. (2018), the authors measured a total of 1301 proteins in plasma in a cohort of 240 healthy individuals aged from 22 to 93 years old with the goal to identify proteins associated with chronological age while avoiding as much as possible the effect of clinically detectable diseases. After multiple adjustments for body mass index and serum creatinine for instance, they assembled a list of 210 age associated proteins. The association of these age-related proteins with several clinical characteristics was analyzed and the authors analyzed how proteome can predict chronological age by designing a proteomic signature of age using an elastic net regression model. Several predictors were built using different subsets of age associated proteins ranging from 76 predictor proteins to only one protein. This first study was later expended by considering a second population sample of almost one thousand participants in the Italy-based InCHIANTI study (https://www.nia.nih.gov/inchianti-study) (Tanaka et al., 2020). The purpose of this second analysis made on a much larger and diverse cohort was to confirm the age-associated proteins reported in the first study, and to uncover their relationship with ARDs. The authors used a two-sample Mendelian Randomization method to study the causal relationship between age-related proteins with ARDs. Interestingly, for some age-related proteins, DNA methylation was shown to partially explain the observed age associations. Another study that showed how proteins that significantly change their expression level with age are also often proteins that directly impact longevity and the onset of ARDs was presented in (Lehallier et al., 2020) where the authors measured the q-value and age coefficient of 529 previously identified aging plasma proteins (103 of which have a HAGR listing). The proteins were analyzed in a plasma proteomic dataset derived from 4263 individuals, using an online software tool. The authors found that approximately 95% of them significantly (q < 0.05) changed their expression level with age. They performed ML modeling and fitted a LASSO linear regression on a plasma proteomic dataset derived from 3301 individuals, finding an ultra-predictive aging clock composed of 491 protein entries. The latter was used to demonstrate, for example, that aerobic exercise trained individuals are predicted to be younger than their actual biological age, compared with physically sedentary subjects. Moreover, they unveiled a multitude of novel aging clocks that are made up of a smaller set of proteins (Lehallier et al., 2020). This has the obvious effect of reducing the costs, making the prediction of patient age logistically simpler and therefore easier to implement on a larger scale.

## 3 Antagonistic hallmarks

### 3.1 Mitochondrial dysfunctions

Mitochondria are dynamic structures well-known for primarily acting as cellular energy generators by producing adenosine triphosphate (ATP) either through mitochondrial oxidative phosphorylation or through anaerobic glycolysis, a second ATP production route in which nicotinamide adenine dinucleotide (NAD+), a small molecule that regulates many biological processes, plays an important role (Aman et al., 2019). Mitochondrial homeostasis and maintenance are unsurprisingly considered as key to health and paramount for healthy aging. Mitochondrial homeostasis and quality are strongly regulated by mitochondrial-autophagy, termed mitophagy, the biological process responsible for the elimination of defective mitochondria. Mitophagy was shown to be important in neurons for the maintenance of neuronal function and to prevent neuronal cell death and pathogenic brain ageing, which are partially caused by an impairment of mitophagy and subsequent accumulating dysfunctional mitochondria. Mitochondrial dysfunction has been recognized for a long time as an antagonistic hallmark of ageing and is an important component of the age-related cellular processes that contribute to the onset of ARDs. For instance, mutations of nuclear- or mitochondria-encoded mitochondrial proteins are known to trigger mitochondrial disorders (Scheibye-Knudsen et al., 2015), while mitochondria-mediated ATP deprivation and oxidative stress are associated to the pathogenesis of cancer and neurodegenerative diseases such as AD and PD. As impairment of mitophagy is common to many age-related neurodegenerative pathologies such as Alzheimer’s disease, ML and AI approaches have been deployed to identify mitophagy modulators that could be used to design novel strategies to improve removal of dysfunctional mitochondria (Xie et al., 2022). In Xie et al. (2022), the authors carried out a computational screening of a large library of natural compounds using both supervised and unsupervised ML approaches to identify new mitophagy-inducing compounds. The workflow utilized vector representations of molecular structures, pharmacophore fingerprinting and conformer fingerprinting to identify two potent mitophagy inducers (Kaempferol and Rhapontigenin) whose activity was tested *in vivo* in nematode and rodent models of AD. Other strategies proposed to focus on the NAD + mitophagy axis. Indeed, the molecular mechanisms of the NAD + mitophagy axis are globally well understood and there is an accumulation of evidence that suggest a correlation between the onset of AD with the depletion of NAD + levels that impacts mitochondrial biogenesis and the clearance of damaged mitochondria. Therapeutic strategies to boost NAD + levels are thus considered as promising treatments against AD and neurodegenerative diseases in general. AI/ML technologies are going to play an important role in this endeavor in areas such as compound screening, lead compound discovery, drug target identification and biomarker development (Aman et al., 2019; Ruixue, et al., 2021).

### 3.2 Deregulated Nutrient Sensing

The highly conserved mechanistic target of rapamycin signaling pathway (mTOR) is the main nutrient sensor and works as a key controller of cellular metabolism and cell organization. mTOR complexes (TORC1 and TORC2) have multiple interactions with various intracellular molecules, mainly the Insulin Growth Factor (IGF) and AMP-activated protein kinase (AMPK) contributing to coordinate a plethora of cellular processes including gene transcription, translation, autophagy, as well as cell metabolism (Bjedov and Rallis, 2020). mTOR activation is critically involved in the autophagy process, a degradation system that mediates the breakdown of major macromolecules (lipids, polysaccharides, and proteins). This process is constantly active and permits to maintain the physiological activity of cells and their survival also in states of metabolic imbalance. Its deregulation strongly contributes to biological effects occurring in aging and chronic diseases of elderly (Lamming and Bar-Peled, 2019; Bjedov and Rallis, 2020). Interventional strategies (genetic, pharmacological, and behavioral) are known to act *via* decreasing mTOR activity, suggesting that its hyperactivation supports the age-related functional failure (Lamming and Bar-Peled, 2019). As a definite dietary intervention that effectively increases healthy lifespan has not been delineated so far, caloric restriction (CR) regimen and alternative nutritional strategies have been reported as valuable strategies to promote healthy aging in animal models, also suggesting a similar effect in humans (Flanagan et al., 2020). Several drugs and other compounds have been shown to act as CR mimetics with various mechanisms, being directly or indirectly associated with mTOR-mediated autophagy regulation (Chung and Chung, 2019; Stead et al., 2019).

Bioinformatics combined with ML algorithms is commonly used to investigate the relationships between autophagy/apoptosis and aging. The consensus supports the role of numerous proteins and genes as predictors of aging-relatedness (Kurz et al., 2008; Kerber et al., 2009). For instance, using supervised ML systems, the gene AKT1 (associated with apoptosis) was revealed as being age-related with high probability (Xu et al., 2002). In addition, experiments on mice revealed that if a cellular protein has an influence on CDK1 (involved in apoptosis), then it is probably linked to aging-process (Xu et al., 2012; Fabris and Freitas, 2016). An autophagy flux sensor, named red-green-blue-LC3 (RGB-LC3) was developed to detect the different footsteps of autophagy progression and the deregulation of this process at different levels (Kim et al., 2020). In addition, different computational methods have been applied for the mathematical modeling of the core regulatory machine of autophagy (Sarmah et al., 2021). The integration of different data types can widen our knowledge of the molecular mechanisms governing autophagy. This could be helpful in the context of the development of targeted therapies (Sarmah et al., 2021).

### 3.3 Cellular senescence

**FIGURE 5:**
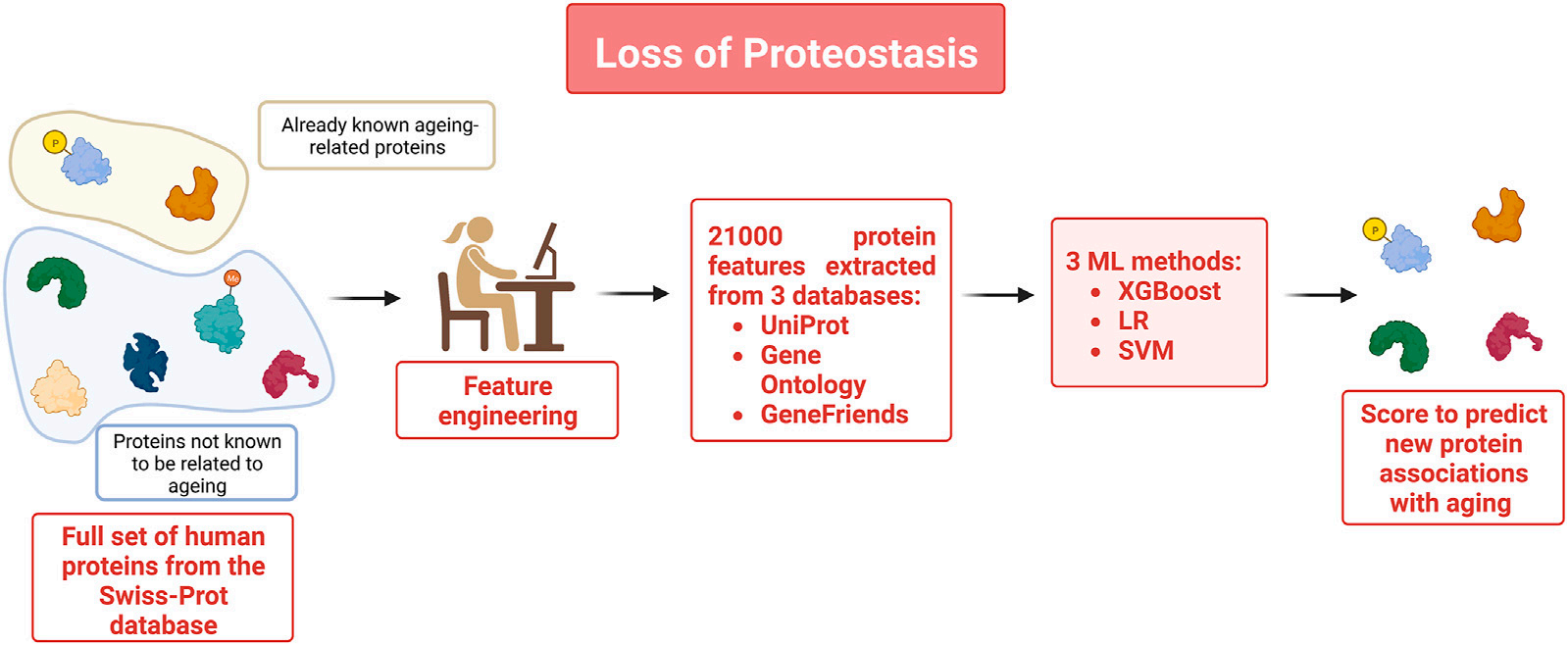
To identify senescent cells, this study relied on the fact that senescent cells elicit a very specific morphology to generate a set of features using cell morphology images obtained by phase-contrast microscopy. The set of 50 × 50 pixels images were used to train a CNN as a classifier that could distinguish senescent cells from healthy cells with a greater accuracy than classical ML methods (SVM, RF, and LR).

Cellular senescence is defined as a condition in which a cell can no longer proliferate. The accumulation of senescent cells is one of the most important processes in aging (Silva-Álvarez et al., 2019). Senescent cells are in the G1 phase of the cell cycle, and even if they are not responsive to external stimuli, they are metabolically active and can modify gene expression. Senescent cells can be found in various tissues affected by various diseases (osteoarthritis, pulmonary fibrosis, atherosclerosis, Alzheimer’s disease, liver fibrosis and cancer) and play an important role in tumor genesis, as demonstrated in mice that undergo senescence (Krizhanovsky et al., 2008; Naylor et al., 2013). Senescent cells are known to have a unique morphology that can be easily identified, and cell morphology images obtained by phase-contrast microscopy contain numerous biological data such as cellular identity and status that can be used as input for a morphology-based identification system that could be utilized to distinguish senescent cells from others. In this context, endothelial cells have attracted interest because they serve many functions in homoeostasis and are involved in the pathology of age-related diseases through cellular senescence. Recent studies (Kusumoto et al., 2018) have proposed to use convolutional neural networks (CNN) to identify endothelial cells derived from pluripotent stem cells, using phase-contrast microscopy images. This CNN system was later adapted by the same research team to identify senescent cells (Kusumoto et al., 2021). In this study, the images to be used as input data were obtained by inducing cellular senescence in human umbilical vein endothelial cells with hydrogen peroxide (H2O2) and camptothecin (CPT). 50 × 50 pixels of input datasets were prepared at the single-cell resolution level from phase-contrast images acquired under each condition. The final number of pictures was 92,242 for H2O2-induced senescence, 41,207 for H2O2 control, 134,097 for CPT-induced senescence, and 64,535 for CPT control. The images were then analyzed in a network to predict them as senescence or control. The predictions were compared with predetermined answers, and weights were optimized to train the CNN. The non-linear prediction of the CNN provides a binary output, meaning that the CNN classified cells as senescent or control (*cf.* Figure 5). These results were shown to be superior to the ones obtained with three other ML methods (SVM, RF, and LR). In that case, the features needed to generate the inputs were extracted using Histograms of Oriented Gradients, a commonly used feature descriptor (Dalal and Triggs, 2005).

The possibility to properly characterize and identify senescent cells is an important step toward the development of therapeutics that can be used to remove them from host tissues. The most well-known of these compounds are called “senolytics”, a new family of natural compounds such as Dasatinib and Quercetin, that could be useful to neutralize senescent cells (Song et al., 2020). Several companies have started the development of novel therapeutic drugs in this area with AI, using different algorithms for the so-called Life Extending Medicine (Dolgin, 2020). The company Dorian Therapeutics developed a new class of therapeutics that can rejuvenate cells and tissues: the “senoblockers”. They focused on the function of Usp16 that contrasts the self-renewal and senescence pathways, switching the entire genetic program of the cells into a more useful expression profile. In human tissues, overexpression of Usp16 reduces the expansion of normal fibroblasts and post-natal neural progenitors, while downregulation of Usp16 partially rescues the proliferation defects of fibroblasts and expands the stem cell compartment in blood, mammary epithelial tissue, and brain. Usp16 is known to have an important role in the accelerated aging observed in people with Down’s Syndrome (Adorno et al., 2013). Another company, Rubedo Life Sciences, is developing a novel method using small molecules to selectively target and clear senescent cells from aged or pathological tissues using the platform ALEMBIC. Their new class of senolytic prodrugs promises capabilities such as targeting selectively and safely specific senescent cell types in multiple tissues to treat age-related diseases in geriatric people (Doan et al., 2020).

It will interesting to see how these different techniques will be combined into an end-to-end biomarker development, target identification, drug discovery and real-world evidence pipeline that may help accelerate and improve pharmaceutical research (Zhavoronkov et al., 2019a).

## 4 Integrative hallmarks

### 4.1 Stem cell exhaustion

The regenerative capabilities of the tissues depend on the pool of stem cells, known to be able to differentiate into different predefined cell types. The newly differentiated cells play an important role for tissue and organ maintenance. It is known that these pools of stem cells tend to decrease over time and finding ways to replenish exhausted stem cell pools within tissues is a major axis of stem cell research and regenerative medicine. Stem cell exhaustion is thus intrinsically associated with aging. The decline of the regenerative potential of the tissues is a multifactorial process impacted by the accumulation of DNA damages (Rossi et al., 2007), telomere shortening (Flores and Blasco, 2010) and excessive proliferation of progenitor cells (Rera et al., 2011). There are three ways, ML/AI technologies may play a major role in this context. Firstly, AI/ML can be deployed to help to elucidate unknowns surrounding the mechanisms behind stem cell fate decision and cellular specialization. Secondly, the capabilities of ML/AI to handle classification tasks where multiple features and non-linear relationships between them must be considered can be highly beneficial for stem cell classification. Thirdly, ML/AI technologies can be used for the design of new systems for cellular engineering in the context of the development of novel stem cell therapies.

#### 4.1.1 Stem cell fate decision and cellular specialization

Depending on their self-renewal and differentiation (specialization) capabilities, stem cells are classified into different categories. The most famous are probably pluripotent stem cells, such as embryonic stem cells (ESCs), which can give rise to every cell type in the formed body, but not the placenta and umbilical cord (Labusca and Mashayekhi, 2019). Studies demonstrated that adult skin tissues contain cell populations with pluripotent characteristics (Chunmeng and Tianmin, 2004). On the other hand, multipotent stem cells, for example, can develop into more than one cell type but are more limited than pluripotent stem cells. Multipotent stem cells from hair follicle and non-follicular skin for instance are found to have the differentiation capacity to generate multiple cell lineages. Other examples of multipotent stem cells are adult stem cells and cord blood stem cells. Sobhani et al. (2017). Observations show that pluripotency (or multipotency) state maintenance which is critical for tissue regenerative abilities is a function of the external environment of the stem cells. A dynamical balance between environmental factors and cellular signals help to preserve the tissue regenerative capacity of stem cells. Stem cell differentiation into a specific cell lineage is often induced by an alteration of this dynamical balance by external perturbations which activate or inhibit biological pathways. This impacts the transduction signals received by transcription factors (TFs) which form highly connected gene regulatory networks (GRNs) within the nucleus (Thomson et al., 2011; Tantin, 2013) and ultimately act as master regulators of the stem cell fate decision (Iglesias-Bartolome and Gutkind, 2011; Tsai and Hung, 2012). Thus, a realistic description of how stem cell fate decision operates should consider not only the GRNs localized inside the nucleus but also the network of signaling pathways located and operating inside the cytoplasmic compartment which transmit signals received from the stem cell environment.

The fact that stem cell fate decision can effectively be controlled through activation or inhibition of TFs was demonstrated in (Takahashi and Yamanaka, 2006) where the authors used AI algorithms to identify key regulators of stem cell fate decision. Thanks to the discovery of key regulatory TFs, they were the first to generate Induced Pluripotent Stem Cells (iPSCs). Another study by Dunn and others used computational modeling to elucidate the dynamics of the GRNs controlling the fate of self-renewing mouse Embryonic Stem Cells (ESCs) (Dunn et al., 2019). They were able to show that a common deterministic gene regulation program might be sufficient to govern the maintenance and induction of naïve pluripotency.

Since then, the field of cellular specialization, which studies stem cell differentiation continued to study the molecular basis of stem cell fate decision as much behind the mechanisms of differentiation remain to be clarified. Regarding the role played by GRNs in stem cell fate decision, a recent study (Gheorghe et al., 2019) showed how to develop the ChIP-eat model, combining computational TF binding models and chromatin immunoprecipitation followed by sequencing (ChIP-seq) to automatically predict direct TF-DNA interactions. Other studies focused on developing a comprehensive evaluation of state-of-the-art algorithms for inferring GRNs from single-cell gene expression data. In (Pratapa et al., 2020), the authors developed BEELINE to use synthetic networks with predictable cellular trajectories and curated Boolean models to evaluate GRN inference algorithms’ accuracy.

From a therapeutic perspective, understanding stem cell decision and specialization would open the doors to tremendous long-term opportunities. An important area of research aims at developing methods to predict the behavior or function of cells produced using methods from synthetic biology. Those cells do not mimic *in vivo* identity but are able to perform specific functions, as cell fate reprogramming is often performed by constant overexpression of specific TFs. However, this process can be unreliable and inefficient. Therefore, approaches based on mathematical analysis and computational methods are expected to be the way to go for the future developments of the discipline (Del Vecchio et al., 2017). For example, in Stumpf et al. (2017) the authors propose to study cell fate using a framework where stem cell differentiation is modeled as a non-Markov stochastic process. Another example is presented in Jones et al. (2020), where the authors generated an experimental lineage tracing dataset with 34,557 human cells continuously traced over 15 generations. In this case, the CRISPR/Cas9-based gene editing approach Jiang and Doudna, (2017) combined with AI can be highly effective.

#### 4.1.2 Stem cell classification

AI systems are used to identify and analyze genes involved in stem cell differentiation and specialization (Haque et al., 2017). An example of such system is the GCTx-TFome, which was used to discover 240 previously unreported TFs involved in ESC differentiation. This system operates by performing large computational screening of the human transcriptome (Ng et al., 2021). CNN is another example of deep learning architecture which was originally a very popular tool in computer vision, given its efficiency in modeling two-dimensional data (LeCun et al., 1999). The development of CNN enables the automation of the cell type identification from phase-contrast microscope images without molecular labeling. The objective is to develop a program that can judge medical conditions as accurately as a physician (Kusumoto and Yuasa, 2019). As discussed above, CNN has been used to create an automated method to identify endothelial cells derived from iPSCs without the need for immunostaining or lineage tracing (Kusumoto et al., 2018).

Another approach to identify stem cells is based on scRNA-seq data. AI can be used to identify stem cells from scRNA-seq data to search for peculiarities of stem cells in the query data. RNA sequencing (RNA-seq) is a genomic approach for the detection and quantitative analysis of messenger RNA molecules in a biological sample and is useful for studying cellular responses (Haque et al., 2017). More recently, (Gulati et al., 2020), developed CytoTRACE, a computational framework based on the simple observation of transcriptional diversity, the number of genes expressed in a cell, and obtained promising results for the prediction of cellular differentiation states from scRNA-seq data.

#### 4.1.3 Cellular engineering

The goal of cellular engineering is to design new therapeutics for patients leveraging the knowledge of the mechanisms behind stem cell fate decision and cellular specialization. In this context, AI is used to evaluate the quality of the engineered cells and to suggest improvements. One example is CellNet (Cahan et al., 2021), a network biology platform that assesses the fidelity of cellular engineering more accurately than any other existing methodology and generates hypotheses for improving cell derivations.

AI can be used to understand the molecular state of a cell in a tissue or within a population, which usually varies stochastically in response to its environment. In many situations, it may be difficult to quantify or even identify the different costs and benefits of a particular response for a cell, particularly for cells in multicellular organisms (Perkins and Swain, 2009). Trajectory inference (TI) is the computational task of determining the position of single cells on temporally regulated biological processes. This method can allow, for example, to study linear tracing with higher accuracy and fidelity. This method is relatively new, and much work will be done to develop it and make it more effective. When designing a system for TI, one needs to consider different indicators such as accuracy, usability, and stability (Saelens et al., 2019). All these features can offer the possibility to define new therapeutics to decrease stem cell exhaustion and allow to tailor the therapy to the specific needs of the patients.

### 4.2 Altered intercellular communication

The coordination of biological processes and activities between the different cells of an organism occur through the extracellular environment and is a necessary condition for the maintenance of homeostasis within any pluricellular organisms. In this context, cell-cell interactions (CCIs) across the various cell types and tissues are regulated through different types of molecules, including ions, metabolites, integrins, receptors, junction and structural proteins as well as ligands and other secreted proteins located in the extracellular matrix (Armingol et al., 2021). These molecules intervene to regulate CCIs in different ways with some like cell adhesion proteins supporting structural CCIs while other factors such as hormones, growth factors, chemokines, cytokines and neurotransmitters act as ligands to mediate cell–cell communication (Armingol et al., 2021).

Aging is associated with alterations of CCI characterized by a deregulation of endocrine, neuroendocrine, or neuronal signaling, a decrease of immunosurveillance as well as an alteration of the composition of peri- and -extracellular environment. Alterations of the CCI also appear with the emergence of senescent cells which utilize three means of intercellular communication known as classical, emerging, and non-classical (Fafián-Labora and O’Loghlen, 2020). The “senescence-associated secretory phenotype” (SASP) is considered as the classical mean of CCI of senescent cells and can result in both beneficial and detrimental effects according to the trigger factors and the context present when senescence is induced. In general, senescence reactivates the expression of multiple pro-inflammatory genes in many different cell types with a profound alteration of SASP composition enriched in pro-inflammatory cytokines and soluble factors such as IL-6, IL-8, membrane cofactor proteins and macrophage inflammatory. Such molecules tend to promote proliferation, angiogenesis, and inflammation, both in autocrine and paracrine manners (Lopes-Paciencia et al., 2019).

Recent results were obtained on the role of extracellular vesicles as novel SASP components as well as non-cellular metabolites and ions. The new emerging picture supports the hypothesis that a simultaneous combination of all these components may contribute to the deleterious effects associated with senescence (Lopes-Paciencia et al., 2019; Fafián-Labora and O’Loghlen, 2020). One of the major changes which takes place with aging is a chronic and systemic, low-grade dysfunctional inflammation, known as inflammaging, where most of the inflammatory factors involved are also part of the SASP. Thus, the SASP is a primary mediator of the detrimental effects of senescent cells, contributing to the development of a state of chronic, low-grade inflammation which is characterized by high levels of circulating cytokines and increased immune infiltration associated with an increased risk of diseases with age (Lopes-Paciencia et al., 2019). Strategies to eliminate senescent cells and/or to modulate SASP have been investigated with the hope that such approaches could bring therapeutic benefits (see also Section 3.3). One of the main focuses of this research is the development of a new class of drugs referred to as senotherapeutics. This class of drugs consists of two members: senolytics and senomorphics. While senolytics are small molecules that can selectively kill senescent cells through apoptosis, senomorphics drugs have the capacity to at block SASP thus reducing the senescent burden of the cells.

The promising capabilities of senotherapeutics to improve health and contribute to the substantial extension of healthy lifespan have already been reported, even if the results currently available showing the effects of these drugs were obtained from preclinical animal studies, which raise legitimate concerns about a potential underestimation of the side effects resulting from a long-term use and chronic administrations. Regarding senomorphics agents, the molecular identification of SASP factors with a detailed characterization of the different pathways responsible for the expected outcomes may enable future interventions in different tissues (Lagoumtzi and Niki, 2021). In a recent study, a drug-screening system for cellular senescence using a pre-trained CNN identified four compounds (terreic acid, PD-98059, daidzein, and Y-27632 2HCl) with the potential capability to repress senescence *in vitro* (Kumari and Jat, 2021). Through the analysis of transcriptome data, these compounds showed a) anti-inflammatory effects *via* the suppression of NF-KB signaling, a pathway that plays a central role in inflammation and b) the appearance of SASP, indicating that these compounds could be strong candidates for the design of new treatments against ARDs (Kusumoto et al., 2021).

Finally, many efforts have been undertaken to develop guidelines on stem cell applications because the field is still in its infancy. For instance, in (Cahan et al., 2021) the authors described four major applications of stem cell biology (cell typing, lineage tracing, trajectory inference, and regulatory networks) with a detailed overview of the future challenges to be addressed in the near future.

## 5 Longevity medicine: Translating and applying the hallmarks of aging into the practice

Despite the currently well-established scientific and technological foundations for clinically guided longevity medicine, there is still a large gap between the geroscience and AI-based tools. This translational bridge is challenging, since this new burgeoning medical discipline is of a distinct character, shaped by multi- (virtually all disciplines of the clinical medicine, including genetics, radiology and pathology, etc.) and interdisciplinarity (AI, computational science, gerontology, gerosciences, engineering, etc.), with strong roots in internal medicine dealing with the complexity of co and multimorbidity (Bischof et al., 2022). Longevity medicine has not yet been officially defined by a central medical body, but expert recommendations suggested that longevity medicine is an AI-driven precision medicine, guided by biological age determination with deep aging clocks (Zhavoronkov et al., 2019a). The formal definition might be further enriched by the core goal of longevity medicine, which is to establish and restore the biological age of an individual at each specific point of time to the biological age of the optimal individual performance (Bischof et al., 2022). Longitudinally and cumulatively, this leads to mitigation and ideally also elimination of risks of age-related and overall morbidity. Therefore, the main focus of longevity medicine is to prolong the life lived in good health, both physically and mentally, ergo: extension of the healthy lifespan and not solely the health span (simple prevention) or solely the lifespan (reactive medicine/sickcare) (Bischof et al., 2022).

The release, in 2018, of the first deep aging clocks was a crucial milestone for longevity medicine because it provided clinicians with a wide range of new possibilities to vigilantly track the progress of a patient’s/individual’s biological age, based on various modalities, e.g., hematological tests, methylation, metabolomic, microbiome, etc. (Zhavoronkov et al., 2019b). Since the progress has been tremendously rapid, most medical professionals are not exposed to the foundations of longevity medicine, nor to its latest fueling scientific and AI sources. Educational resources adapted to clinicians are scarce, with solely one Center for Medical Education offering an accredited course of Longevity Medicine for Physicians (Bischof et al., 2021). However, the autodidactic efforts are increasing, therefore academic programs started to implement healthy aging in the educational curriculum to equip healthcare professionals with the necessary understanding of aging research. Soon, the Healthy Longevity Medicine Society (https://hlms.co/) will coordinate the development of recommendations and guidelines that will allow to credibly validate and further establish biomarkers of aging, while also educating medical clinicians on how to implement the knowledge in their work with the patients.

## 6 Conclusion

In the last few years, we have witnessed many AI-enabled technological and biological companies initiating collaborations to use a variety of algorithms to identify lifestyle characteristics that influence how individual age as well as to develop drugs and therapeutic medicine to counteract the deleterious effects of aging and ARDs (Dolgin, 2020). Moreover, AI technologies also hold great promises to study the molecular state of tissues, organs, or cells in response to physical or chemical change in the environment.

When applying ML/AI in the field of aging research, one should keep in mind one major characteristics of this phenomenon which is that, rather than being a localized event, aging is an intrinsically systematic process. The systemic characteristic of aging is well illustrated by the hallmarks discussed herein which despite being of different nature are also highly mechanistically intertwined. The systemic nature of aging can be seen as the result of the hierarchical organization of living systems (Han et al., 2004; Buescher et al., 2012; Nicolas et al., 2012). The human body in particular is a multi-level complex system consisting of billions of independent cells which form different types of tissues, organs and regulatory systems. Dysfunctions affecting even a restricted number of biological processes within some of the cells of one or several organs will often propagate to all parts of the body (Vanhaelen, 2015; Vanhaelen, 2018). The network theory of aging was specifically designed to overcome the reductive nature of the first individual dynamical models usually focusing on a restricted set of hallmark-related phenomena. The main purpose of the network theory of aging was to integrate different mechanisms of aging into a common framework to better understand the systemic consequences of their continuous mutual interactions (Kowald and Kirkwood, 1994; Kowald and Kirkwood, 1996; Franceschi et al., 2000; Slijepcevic, 2008). In this context, modern ML/AI can provide valuable modeling tools to investigate how all aging mechanisms work together, and to shape the global aging pattern.

In this overview, we have shown a variety of approaches from the technology and biology field, which can help in the development of biological markers, in the identification of new targets in the cells, in making the drug discovery process more efficient, and in therapeutics aimed at increasing the age expectation. The field of investigation is relatively young, and the early results have already shown the enormous application potential of the new technologies. Therefore, it is not difficult to foresee AI as an essential component for future life and health science research and for the pharmaceutical industry towards new discoveries that could guarantee a healthier and longer life for everyone (Zhavoronkov et al., 2019a; Zhavoronkov et al., 2021).
